# Antimicrobial Resistance Pilot Surveillance of Pigs and Chickens in Vietnam, 2017–2019

**DOI:** 10.3389/fvets.2021.618497

**Published:** 2021-07-08

**Authors:** C. V. Tuat, P. T. Hue, N. T. P. Loan, N. T. Thuy, L. T. Hue, V. N. Giang, Vera I. Erickson, Pawin Padungtod

**Affiliations:** ^1^Department of Animal Health, Ministry of Agriculture and Rural Development, Hanoi, Vietnam; ^2^Food and Agriculture Organization of the United Nations Country Office for Vietnam, Hanoi, Vietnam

**Keywords:** pig, chicken, *Salmonella*, *Escherichia coli*, anti-bacterial agents, drug resistance, public health, livestock

## Abstract

Antimicrobial use (AMU) and antimicrobial resistance (AMR) are a growing public health and economic threat in Vietnam. We conducted a pilot surveillance programme in five provinces of Vietnam, two in the south and three in the north, to identify antimicrobial resistance (AMR) in rectal swab samples from pigs and fecal samples from chickens at slaughter points during three different points in time from 2017 to 2019. *Escherichia coli* (*E. coli*) and non-typhoidal *Salmonella* (NTS) isolates were tested for antimicrobial susceptibility using disk diffusion assay for 19 antimicrobial agents belonging to nine antimicrobial classes and Etest for colistin (polymyxin). Almost all *E. coli* (99%; 1029/1042) and NTS (96%; 208/216) isolates were resistant to at least one antimicrobial agent; 94% (981/1042) of *E. coli* and 89% (193/216) of NTS isolates were multidrug-resistant (MDR). Higher proportions of *E. coli* and NTS isolated from chickens were resistant to all antimicrobial classes than those isolates from pigs. There was a significantly higher proportion of MDR NTS isolates from the southern provinces of Ho Chi Minh City and Long An (*p* = 0.008). Although there were increasing trends of NTS in proportion of resistance to fluoroquinolone over the three surveillance rounds, there was a significant decreasing trend of NTS in proportion of resistance to polymyxin (*p* = 0.002). It is important to establish an annual AMR surveillance program for livestock in Vietnam to assess the impact of interventions, observe trends and drive decision making that ultimately contributes to reducing AMR public health threat.

## Introduction

Antimicrobial use (AMU) and antimicrobial resistance (AMR) are a growing public health and economic threat in Vietnam and countries in southeast Asia (1). *Escherichia coli* (*E. coli*) isolated from pig and chicken farms in Vietnam had extremely high resistance to ampicillin and ciprofloxacin, and high resistance to gentamycin and colistin (2). A similar situation has also been observed in Cambodia (3). A very high proportion of non-typhoidal *Salmonella* (NTS), isolated from meat in the market in Vietnam was resistant to quinolone and beta-lactams (4). Overall, the use of antimicrobials in livestock production and poor biosecurity, especially on small farms, were associated with high levels of AMR in Vietnam (2, 5).

Resistance genes, resistant bacteria and antibiotic residues can spread to humans via direct contact with animals, wastewater, slurry, vegetables, and animal by-products (6, 7). Before the ban of antimicrobial growth promoters in 2018 in Vietnam, the most widely used in-feed antibiotics in chicken production were bacitracin, chlortetracycline and enramycin. In pig production the most used antibiotics were bacitracin, chlortetracycline and florfenicol (1).

The World Health Organization (WHO) has listed five antimicrobial classes of highest priority among the critically important antimicrobials (CIAs); cephalosporins (3rd, 4th, and 5th generations), glycopeptides, macrolides and ketolides, polymyxins, and quinolones (8). A 2019 study in Indonesia, Thailand, and Vietnam identified neomycin, colistin and amoxicillin in pig and chicken feeds, all of which are antimicrobials classified as critically important or highly important in treatment of human infections (9).

In 2017, Vietnam's Ministry of Agriculture and Rural Development (MARD) issued a national action plan for AMR and AMU in livestock and aquaculture in line with the Food and Agriculture Organization of the United Nations (FAO) Action Plan on AMR (10). One of the main activities under this plan was to establish and implement a national programme for the surveillance of AMR in animals and food (11). MARD assigned the Department of Animal Health as the focal agency for AMU and AMR management in livestock. In collaboration with Department of Animal Health, FAO, conducted a pilot surveillance to support the development of the national AMR surveillance programme and establish a baseline that could be used to track AMR level changes. This pilot surveillance was designed to estimate AMR in two commensal bacteria, *E. coli* and non-typhoid *Salmonella* (NTS), in pigs and chickens. We expected that the surveillance would also allow comparison of AMR spatially (between geographical regions) and temporally (between rounds of sampling) in pigs and chickens produced in Vietnam.

## Materials and Methods

The pilot surveillance was conducted in five provinces in Vietnam: three in the north, Hanoi, Hai Phong, Quang Ninh; and two in the south, Ho Chi Minh City and Long An. The first round of surveillance was conducted in August 2017, the second round in February 2019 and the third round in August 2019. The target population was chickens sold at wet markets and pigs at slaughter points.

The National Center for Veterinary Hygiene and Inspection (NCVHI) No.1 in Hanoi, which is an assigned laboratory for AMR surveillance, was assessed for technical capacity and biological material management before conducting the first round of the pilot surveillance using FAO Assessment Tool for Laboratory and AMR Surveillance Systems (ATLASS) in April 2017. NCVHI is also accredited by the ISO/IEC 17025 standard for conducting isolation and identification of *E. coli* and NTS.

For the sample size, we followed the European Food Safety Authority's target sample size of 170 isolates tested for each species (12). Seven pigs and seven chickens slaughter points (either at slaughterhouses or wet markets) with the highest number of animals processed in each region (north and south) were selected. NCVHI staff conveniently selected 25 pig rectal swabs from all holding pens, or 25 chicken droppings from all chicken cages from each slaughter points for sample collection. The total sample size of 350 for each species collected each round was assumed to yield at least 170 NTS isolates if the prevalence of NTS was 50% in pigs and chickens (13). All laboratory tests were performed at NCVHI No. 1 were based on protocols, including reference strains established by Department of Animal Health following FAO regional antimicrobial resistance surveillance and monitoring guidelines (14).

Samples for NTS were stored in a falcon tube containing 10 ml buffered peptone water at room temperature (25°C). Samples for *E. coli* were stored in a falcon tubes and kept cool (4°C) immediately after sampling. All *E. coli* or *Salmonella* isolates were stored at the laboratory, preserved in appropriate condition before conducting the antimicrobial susceptibility testing. Isolates to be tested and quality control strains were revived by using a nutrient agar medium. NTS isolates were not further characterized (serogrouping and serotyping). Antimicrobial susceptibility testing of *E. coli* and NTS isolated from pig and chicken fecal samples were conducted using the Kirby-Bauer disk diffusion test for 19 agents belonging to nine antimicrobial classes. Etest was used to determine resistance to colistin. *E. coli* and NTS isolates were classified as resistant using Clinical Laboratory Standard Institute (15) and European Committee on Antimicrobial Susceptibility Testing (www.EUCAST.org) breakpoints. Reference strains for testing quality control were used, including: *Salmonella typhimurium* American Type Culture Collection (ATCC) 14020 for identification of NTS; ATCC *E. coli* 25922 for identification of *E. coli*; *P. Aeruginosa* ATCC 27853 for cat-ion control; *Enterococcus faecalis* ATCC 29212 for thymidine control in the medium; the ATCC *E. coli* 25922 and the *K. pneumoniae* ATCC 700603 for antimicrobial susceptibility testing. A multidrug resistant (MDR) strain is defined as a strain's non-susceptibility to at least one agent in three or more antimicrobial classes (16). Enrofloxacin was classified as resistant if the zone diameter was ≤ 16 mm. For colistin, an Etest (AB Biodisk, Solna, Sweden), a minimum inhibitory concentration (MIC) assay, was used with the criteria that isolates with MIC > 4 were classified as resistant. Proportions of resistance were compared using the chi-square test.

Significant levels of increasing or decreasing trends over the three rounds of sampling were determined using the score test for trend of resistant odds from round one to round three. A multivariable logistic regression model with slaughter points as a random effect was used to estimate odds ratio (OR) of resistance isolated from different species (pig/chicken), regions (north/south) and rounds (August 2017/February 2019/August 2019). We include rounds in the model to clarify the direction of the trend and differences between sampling rounds. Resistance to at least one agent in each antimicrobial class was used as an outcome variable. All variables were kept in the model to control for each other. All statistical tests were conducted at 0.05 significant level. All statistical analysis was performed using STATA (College Station, TX).

Samples and data were collected by the Department of Animal Health in Vietnam. Information on specific animals was collected from slaughterhouses and wet markets without identifying the owners' personal information.

## Results

A total of 1,050 samples were tested (Table 1). The overall *E. coli* isolation rate was 99% (1042/1050) and 21% (216/1050) for NTS. There was significantly (*p* < 0.001) more NTS isolated from pigs (26%, 136/525) than chickens (15%, 80/525) while *E. coli* isolation rate in both pigs (100%, 523/525) and chickens (99%, 519/525) were not significantly different (*p* = 0.156). The isolation rates for *E. coli* (345/350, 350/350, 347/350; *p* = 0.091) and NTS (64/349, 80/350, 72/350; *p* = 0.336) were not significantly different when comparing the three surveillance rounds.

**Table 1:** Collection of pig and chicken samples during three rounds of antimicrobial resistance surveillance in five provinces in Vietnam, 2017–2019.

	**Province**	**Pig**	**Chicken**
Slaughter points	Hanoi	1	2
	Hai Phong	1	1
	Quang Ninh	1	-
	Ho Chi Minh City	2	1
	Long An	2	3
Animals sampled per slaughter points		25	25
Animals sampled per round		175	175
Total number of samples (three rounds)		525	525
Total number of samples tested		1,050	

**Figure 1:**
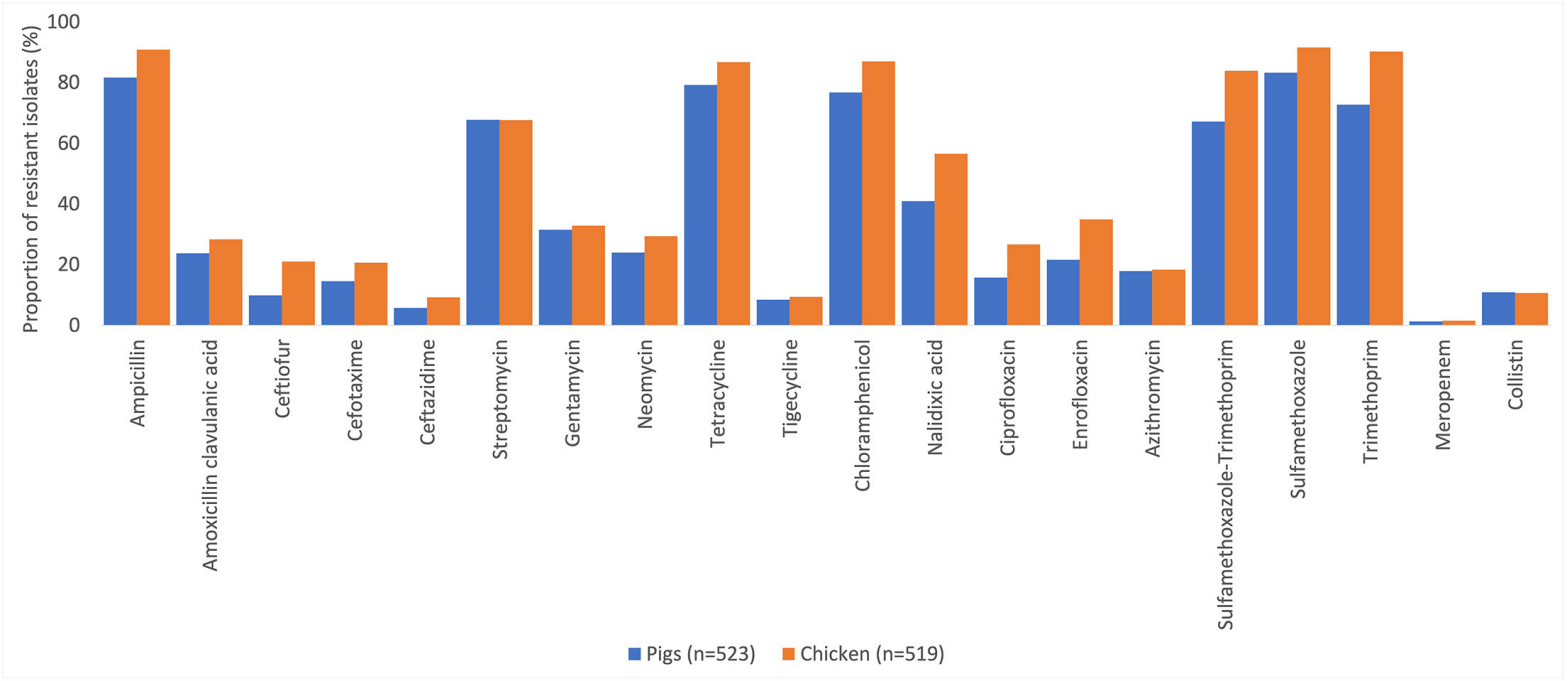
Percentages of antimicrobial resistance in *Escherichia coli* in pig and chicken samples collected from five provinces in Vietnam, 2017–2019.

**Figure 2:**
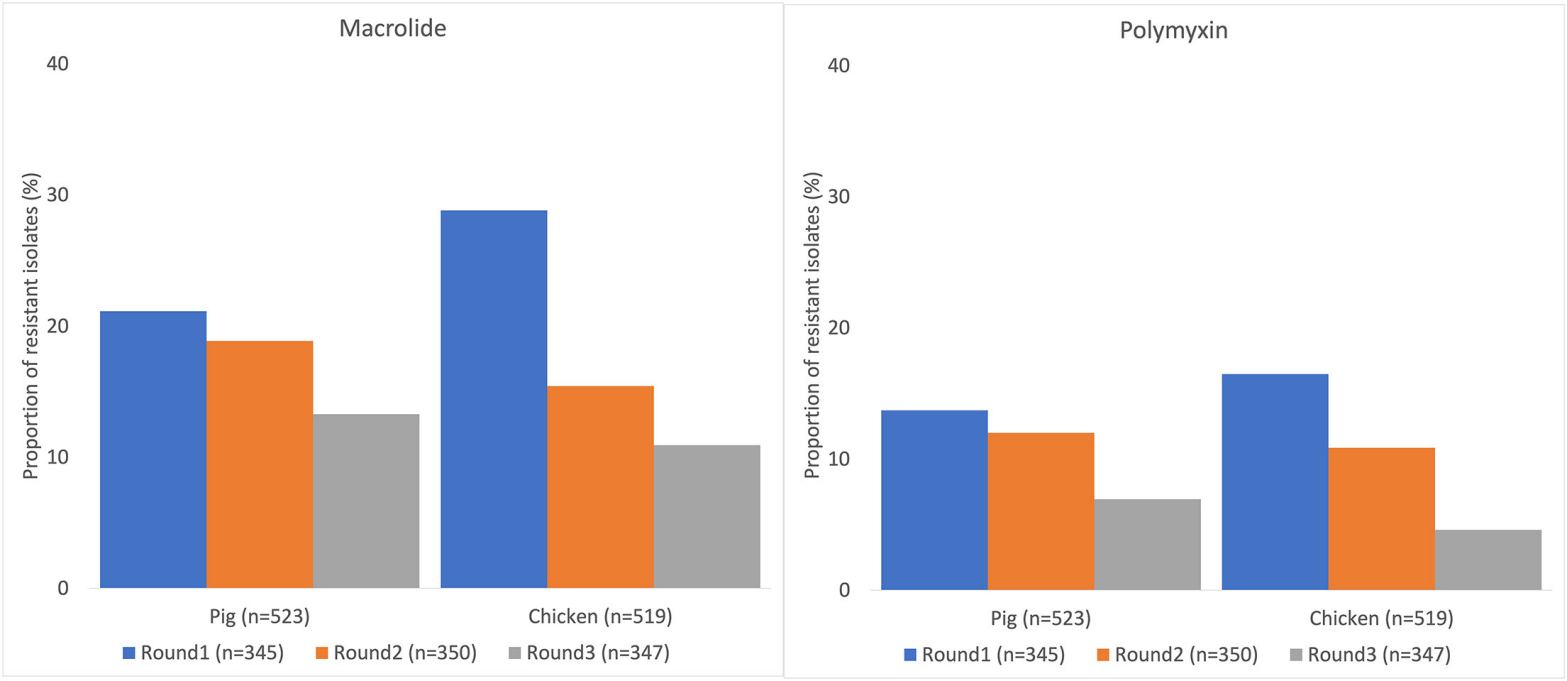
Decreasing trend of *Escherichia coli* resistance to macrolide and polymyxin in pig and chicken samples collected from five provinces in Vietnam, 2017–2019.

Resistance to at least one antimicrobial agent was found in 99% (1042/1050) of *E. coli* and 96% (216/1050) of NTS isolates. MDR was found in 94% (981/1042) of *E. coli* and 89% (193/216) of NTS isolates. Many of the *E. coli* isolates showed extremely high proportions of resistant with more than 70% of isolates being resistance to six antimicrobials tested (Figure 1). A high proportion of *E. coli* isolated from both chickens (95%, 494/519) and pigs (93%, 487/523) were MDR. A higher proportion of *E. coli* isolates from chicken were resistant to all antimicrobial classes, than those isolated from pigs. The proportion of MDR *E. coli* was not significantly different over the three surveillance rounds. The decreasing trend of *E. coli* resistance to macrolide (*p* = 0.001) and polymyxin (*p* < 0.001) is shown in Figure 2. When comparing the two southern and three northern provinces, there was a significantly lower proportion of *E. coli* with resistance to cephalosporin (OR = 0.6, *p* = 0.001) and polymyxin (OR = 0.5, *p* = 0.001) in the south compared to the north (Table 2).

**Table 2:** Association between *Escherichia coli* resistance, species, region and round, Vietnam, 2017–2019.

**Antimicrobial classes**	**Species**		**Region**		**Round**				
	**Chicken/pig**		**South/North**		**2/1**		**3/1**		**Trend *p***
	**OR**	***p***	**OR**	***p***	**OR**	***p***	**OR**	***p***	
Beta—lactam	2.2	<0.001	1.0	0.885	0.7	0.150	0.5	0.001	<0.001
Cephalosporin	1.7	0.001	0.6	0.001	2.0	<0.001	1.1	0.661	<0.001
Aminoglycosides	1.2	0.213	1.0	0.994	0.8	0.002	0.6	0.008	0.011
Tetracycline	1.7	0.006	0.9	0.667	0.3	<0.001	0.3	<0.001	<0.001
Phenicol	2.0	<0.001	1.4	0.049	0.8	0.156	0.9	0.686	<0.001
Fluoroquinolone	1.6	<0.001	0.8	0.087	0.8	0.190	0.6	0.001	<0.001
Macrolides	1.0	0.799	1.0	0.835	0.6	0.012	0.4	<0.001	0.001
Sulphonamides	2.6	<0.001	0.8	0.425	1.0	0.907	1.2	0.646	0.005
Carbapenems	1.2	0.778	0.5	0.193	0.9	0.778	-	-	0.591
Polymyxin	1.0	0.881	0.5	0.001	0.7	0.161	0.3	<0.001	<0.001
Extended-Spectrum Beta-Lactamases	2.2	<0.001	1.0	0.804	2.7	<0.001	3.4	<0.001	<0.001
Resistance to at least one agent	2.3	0.175	1.1	0.821	0.3	0.179	0.4	0.270	0.373
Multi-Drug Resistance	1.5	0.155	1.1	0.853	0.7	0.208	0.8	0.404	0.446

*This table summarizes the between species and between geographical locations analyses; pig and north Vietnam (in bold fonts) were reference levels used. For the temporal analysis (between rounds), rounds 2 and 3 were compared to round 1 of sampling; trends with p values < = 0.05 indicate significant temporal trend over all three rounds using score test*.

**Figure 3:**
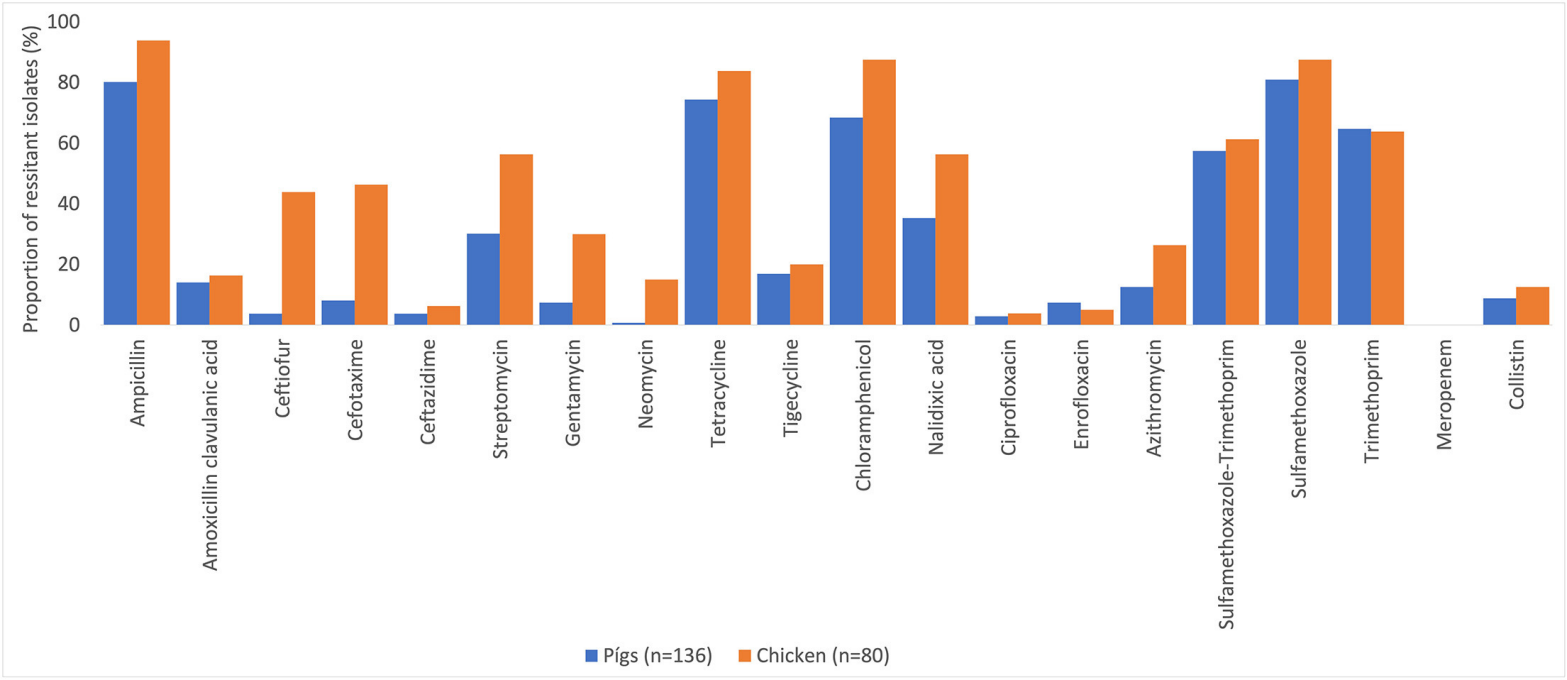
Antimicrobial resistance in non-typhoidal *Salmonella* in pig and chicken samples collected from five provinces in Vietnam for 20 antimicrobials, 2017–2019.

**Figure 4:**
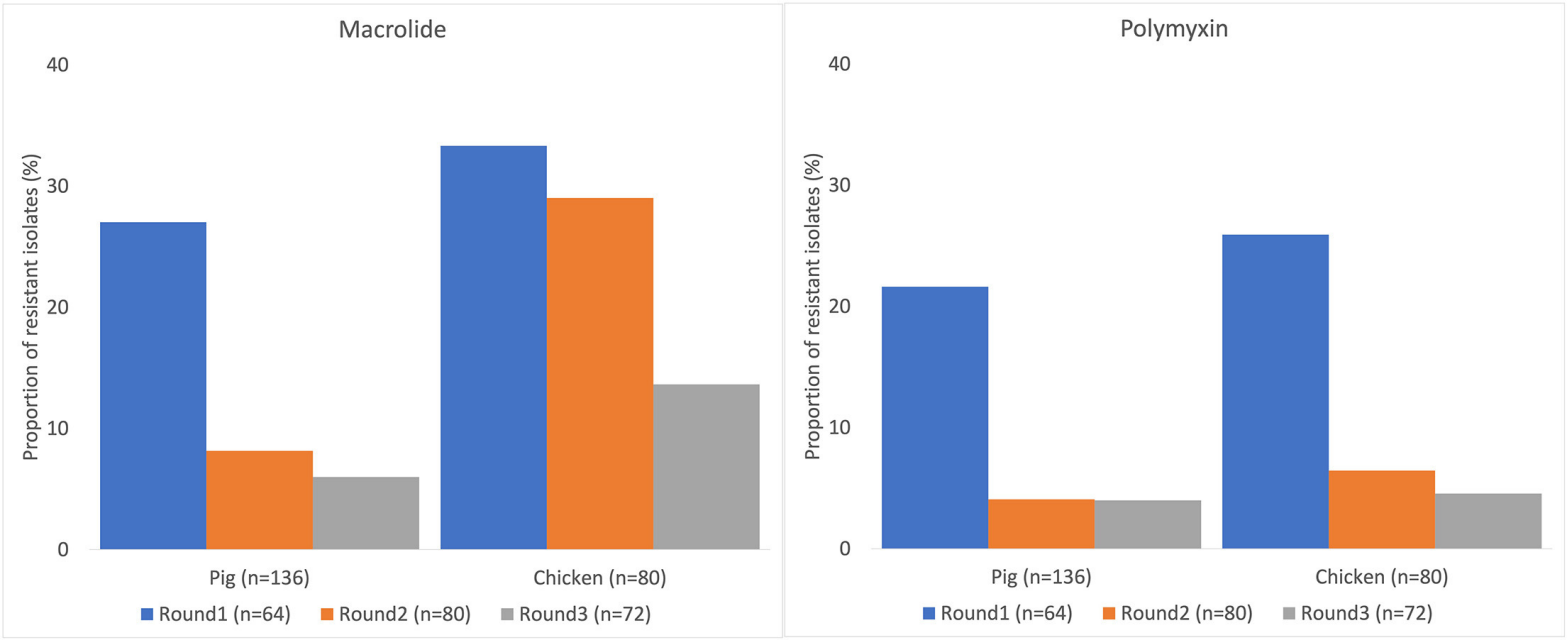
Decreasing trend of non-typhoidal *Salmonella* resistance to macrolide and polymyxin in pig and chicken samples collected from five provinces in Vietnam, 2017–2019.

Many of the NTS isolates showed high proportions of resistance (Figure 3). In NTS isolated from chickens, 95% (76/80) were MDR, while 86% (117/136) of NTS isolated from pigs were MDR. Higher proportions of NTS isolated from chickens were resistant to all antimicrobial classes tested than those isolates from pigs (Table 3). The decreasing trend of NTS resistance to macrolide (*p* < 0.001) and polymyxin (*p* = 0.002) is shown in Figure 4.

**Table 3:** Association between non-typhoidal *Salmonella* resistance, species, region and round, Vietnam, 2017–2019.

**Antimicrobial classes**	**Species**		**Region**		**Round**				
	**Chicken/pig**		**South/North**		**2/1**		**3/1**		**Trend *p***
	**OR**	***p***	**OR**	***p***	**OR**	***p***	**OR**	***p***	
Beta—lactam	4.4	0.008	1.8	0.143	0.8	0.636	0.4	0.077	<0.001
Cephalosporin	22.9	<0.001	0.4	0.010	0.7	0.267	0.3	<0.001	<0.001
Aminoglycosides	4.1	<0.001	0.6	0.294	25.2	<0.001	3.4	0.049	<0.001
Tetracycline	1.7	0.249	1.6	0.226	5.8	0.009	0.4	0.038	<0.001
Phenicol	3.2	0.004	1.8	0.097	5.0	0.002	0.6	0.243	<0.001
Fluoroquinolone	2.9	0.001	3.0	0.001	4.9	<0.001	3.2	0.004	<0.001
Macrolides	2.5	0.016	0.4	0.029	0.5	0.082	0.2	0.003	<0.001
Sulphonamides	1.5	0.387	1.4	0.441	0.9	0.802	0.6	0.308	0.557
Carbapenems	-	-	-	-	-	-	-	-	-
Polymyxin	1.4	0.506	0.7	0.390	0.2	0.004	0.1	0.003	0.002
Extended-Spectrum Beta-Lactamases	18.8	<0.001	0.3	0.026	41.1	<0.001	1.5	0.637	<0.001
Resistance to at least one agent	1.4	0.685	2.0	0.368	-	-	0.2	0.085	0.129
Multi-Drug Resistance	2.9	0.074	3.7	0.008	0.5	0.354	0.2	0.020	0.001

*This table summarizes the between species and between geographical locations analyses; pig and north Vietnam (in bold fonts) were reference levels used. For the temporal analysis (between rounds), rounds 2 and 3 were compared to round 1 of sampling; trends with p values < = 0.05 indicate significant temporal trend over all three rounds using score test*.

The proportion of MDR NTS isolated from pigs and chickens in the southern provinces was significantly higher (OR = 3.7, *p* = 0.008) than in the northern provinces (Table 3). When comparing the two provinces in the south to the three provinces in the north, there was a significantly higher proportion of resistant NTS to fluoroquinolones (OR = 3.0, *p* = 0.001), but a significantly lower proportion of resistance to cephalosporin (OR = 0.4, *p* = 0.010) (Table 3).

The median number of resistant antimicrobial classes in *E. coli* and NTS isolates from chickens was significantly (*p* < 0.001) higher than the median number in pigs (Table 4). There was a significantly higher proportion of MDR NTS isolates from the south (Ho Chi Minh city, Long An; *p* = 0.008) compared to the north (Hanoi, Hai Phong, Quang Ninh). Although there was an increasing trend of NTS in proportion of resistance to fluoroquinolone over the three surveillance rounds, there was a significant decreasing trend of NTS in proportion of resistance to polymyxin. Similar patterns were also observed in *E. coli*.

**Table 4:** Distribution of multidrug resistant isolates collected from pig and chicken samples during three rounds of antimicrobial resistance surveillance in five provinces in Vietnam, 2017–2019.

**Number of classes**	***Escherichia coli***				**Non-typhoidal** ***Salmonella***			
	**Pig (*****n*** **= 514)**		**Chicken (*****n*** **= 515)**		**Pig (*****n*** **= 129)**		**Chicken (*****n*** **= 78)**	
	**No**.	**%**	**No**.	**%**	**No**.	**%**	**No**.	**%**
1	11	2.1	8	1.6	7	5.4	1	1.3
2	16	3.1	13	2.5	6	4.7	1	1.3
3	39	7.6	15	2.9	11	8.5	3	3.8
4	77	15	61	11.8	51	39.5	18	23.1
5	160	31.1	121	23.5	29	22.5	6	7.7
6	129	25.1	147	28.5	20	15.5	10	12.8
7	59	11.5	106	20.6	4	3.1	28	35.9
8	20	3.9	42	8.2	1	0.8	11	14.1
9	3	0.6	2	0.4	0	0	0	0
10	0	0	0	0	0	0	0	0
Median	5		6		4		6	

## Discussion and Conclusion

Both *E. coli* and NTS detected in this study are commensals bacteria that do not cause disease in animals, although some of these strains can cause diseases in human. Overall, this pilot surveillance found almost all *E. coli* and NTS with resistant to at least one antimicrobial tested. A high proportion of *E. coli* and NTS were MDR. Considering resistance to the WHO's CIA, there was a low proportion of *E. coli* and NTS with resistance to third generation cephalosporin (ceftazidime); low to moderate with resistance to polymixin (colistin) and fluoroquinolone (ciprofloxacin); and moderate to high with resistance to macrolide (azithromycin). However, there was a decreasing trend of *E. coli* and NTS resistance to macrolide and polymyxin, shown by both regression and trend analysis, after the 2018 ban of antimicrobial growth promoters. A variety of resistance characteristics between *E. coli* and NTS were identified between the northern and southern provinces of Vietnam.

The prevalence of NTS isolated from pigs (26%, 136/525) and chickens (15%, 80/525) were much lower than the NTS prevalence identified at pig (65%) and chicken (31%) farms (13), and in pork (70%) and chicken meat (70%) sold at the market (4). However, the lower prevalence may have resulted from using individual samples collected from many different sources of animals at slaughter points as opposed to using environmental samples from farms or markets. Despite the lower prevalence, NTS was found in this study and could be potentially be transmitted to humans and cause disease via under cooked food. The proportion of MDR *Salmonella* in Vietnam detected in this study was much higher than those reported earlier in Vietnam (17), Thailand and Cambodia (18). We currently do not have any explanation for this difference, but further analysis comparing antimicrobial consumption among these countries may shed some light on this issue. *Salmonella*, with resistance to third generation cephalosporin, one of WHO highest priority CIAs, was reported earlier in Vietnam with an increasing proportion of resistance from <1% in 2015 to 4% in 2018 and up to 22% (48/216) in this study. Similarly, resistance to fluoroquinolone, another WHO priority CIA, in this and more recent studies, is much higher than earlier reported (4, 13).

We found the proportion of *E. coli* with resistance to colistin in both pigs and chicken (11%, 112/1,042) was lower than other studies (22–24%) had reported earlier (2), corresponding to the lower proportion of Extended-Spectrum Beta-Lactamases (ESBL) producing *E. coli* also found in this study. A study in Vietnam published in 2018 showed a high occurrence of ESBL producing *E. coli* in both pig farmers and pigs that could pose a risk of transmission of these bacteria from pig farms to the community (19).

This pilot surveillance study had several limitations. The model for the sample size was from the European Food Safety Authority that stated the optimal sample size should be 170 positive isolates of *E. coli* and NTS for each bacterium in each animal species. However, we only found 80 positive NTS isolates in chicken, which is arguably not a strong enough sample size to evaluate resistance in NTS. We also assumed that the 25 samples from each slaughter point would represent animals from 25 different locations. However, animals could not be traced back to the farm of origin; this could have led to clustering of samples from the same farms. Fecal samples were collected from conveniently selected droppings from cages in the wet markets. The microbial status of the bird cages may have impacted the results due to inadequate cleaning and disinfection. Although using the disk diffusion test is an affordable method to assess resistance, this method does not provide the MIC to allow for subsequent analysis, especially when different interpretive criteria are used or the criteria change (14). Further typing of NTS found in this study can clarify the risk poses.

In conclusion, the observed dynamic of resistance of *E. coli* and NTS to many antimicrobials warrant the development of a national AMR surveillance programme to monitor the situation and support AMU management policy development. As our findings differed between the two geographical areas, management may need to be customized for the northern and southern areas of the country. The decreasing trend of resistance to WHO's highest priority critically important antimicrobials detected by the surveillance programme can also be used to strengthen the implementation of existing AMU management policy such as banning of antimicrobial growth promoters and advocate for further usage reduction policy such as banning of prophylaxis use. AMR is a public health concern, requiring cross-sectoral collaboration. From a public health perspective, it is especially important to monitor WHO's highest priority critically important antimicrobial classes in animals as well as in humans. We recommend that a national AMR surveillance program for livestock be established in Vietnam. Annual surveillance makes it possible to assess the impact of interventions, observe trends and drive decision making that ultimately contributes to reducing the public health threat from AMR. This antimicrobial resistance data in livestock, generated by the Vietnam Department of Animal Health, has contributed to the global antimicrobial resistance surveillance database and can be used for joint analysis with human health surveillance data under the One Health approach. Future One Health surveillance design, involving human health, animal health and the private sector can be developed through the identification of appropriate levels of collaboration, depending on the expected positive impacts on the value of surveillance (20). The multi-sectoral AMR surveillance, leading to the harmonization and combination of data from different sources, could improve knowledge on transmission routes and risk factors related to AMR in human, animal and environment (21). Specifically, the AMR surveillance programme can be included under the next phase of the national action plan for the management of AMU and monitoring of AMR 2021–2025. The national action plan would provide a legal basis for mobilizing resource from central and provincial authorities to support the surveillance activities and ensure sustainability of the surveillance programme.

## Data Availability Statement

The raw data supporting the conclusions of this article will be made available by the authors, without undue reservation.

## Ethics Statement

Ethical review and approval was not required for the animal study because The surveillance was conducted by a national competent authority for animal health.

## Author Contributions

CT, NT, LH, VG, and PP conceived and designed study. CT, PH, and NL conducted field sample collection and laboratory testing. CT, PH, NL, VE, and PP contributed to data analysis. CT, VE, and PP contributed to writing and editing the manuscript. All authors contributed to the article revision and approved the submitted version.

## Conflict of Interest

The authors declare that the research was conducted in the absence of any commercial or financial relationships that could be construed as a potential conflict of interest.
